# P-798. Urine-Based Expansion of a CRISPR Diagnostic Platform for Antimicrobial Resistance and Bacterial Identification

**DOI:** 10.1093/ofid/ofaf695.1008

**Published:** 2026-01-11

**Authors:** Bhrus Sangruji

**Affiliations:** Broad Institute, Somerville, MA

## Abstract

**Background:**

Urinary tract infections (UTIs) are among the most common bacterial infections globally, yet their diagnosis is often delayed by reliance on culture-based methods. These delays contribute to the misuse of empiric antibiotics and fuel antimicrobial resistance (AMR). The BADLOCK (Bacterial and AMR Detection by SHERLOCK) platform, a CRISPR-Cas13a-based diagnostic system originally developed for bloodstream infections, was adapted here for rapid detection of uropathogens and AMR genes directly from urine.

**Figure 1.**
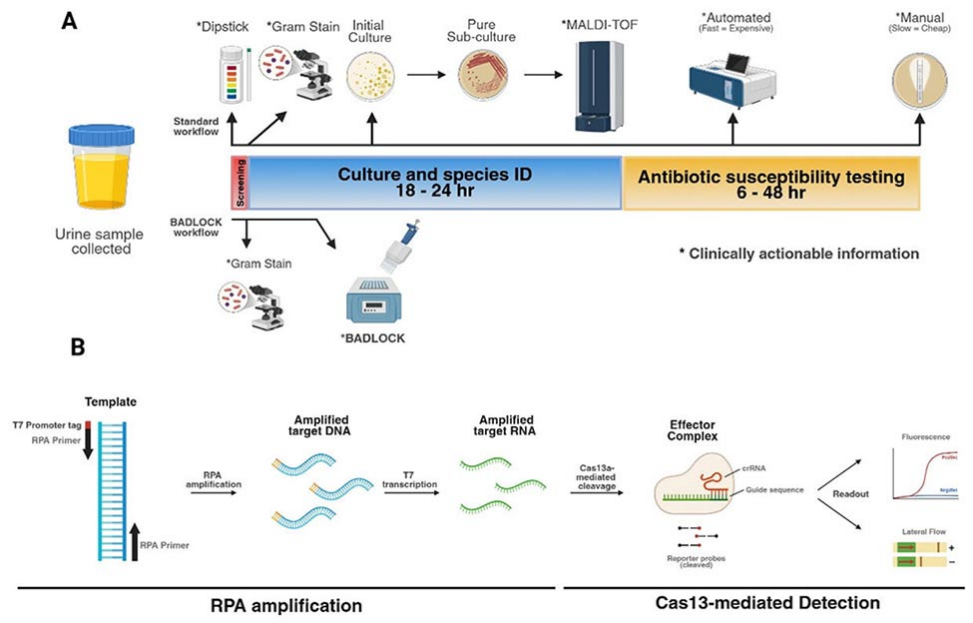
Integration of BADLOCK into UTI Diagnostic Workflow: From Sample Collection to CRISPR-Based Detection BADLOCK workflow enables faster and less resource-intensive detection of urinary tract infections compared to standard diagnostics. (A) Schematic comparing the conventional diagnostic workflow for urine samples (top) with the BADLOCK workflow (bottom). While standard methods require multiple sequential steps and complex instrumentation, BADLOCK provides clinically actionable results within ∼1.5 hours using minimal resources. (B) Molecular mechanism of the SHERLOCK-based BADLOCK platform. Target genes are first amplified via isothermal RPA using primers containing a T7 promoter. The resulting DNA is transcribed into RNA, which is recognized by a CRISPR/Cas13a effector complex. Upon target binding, Cas13a mediates trans-cleavage of reporter probes, generating a detectable signal through fluorescence or lateral flow.

**Figure 2.**
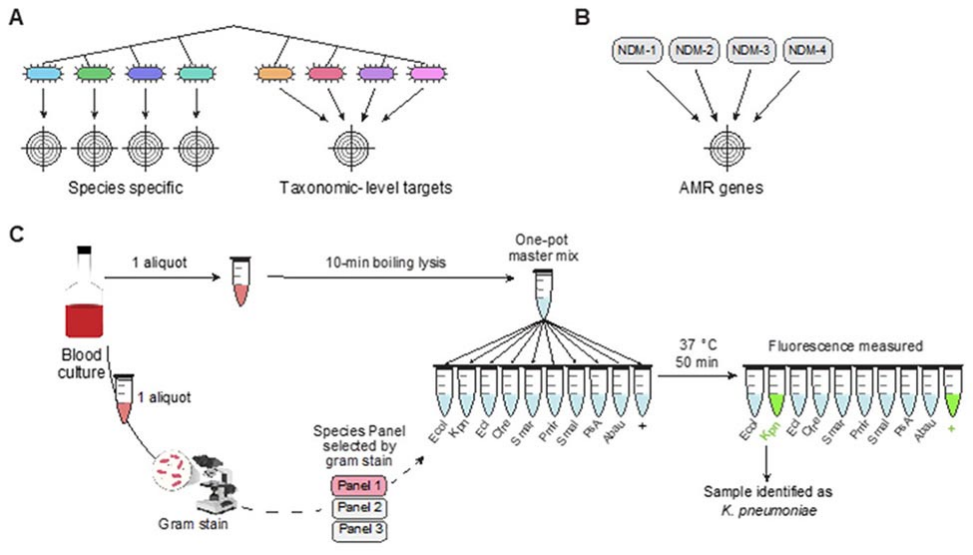
BADLOCK Workflow for Rapid Species and AMR Gene Detection Target selection strategy and overview of the BADLOCK diagnostic workflow. (A) Targets are designed to detect either individual species or closely related bacteria within the same genus that share similar clinical management strategies. (B) Antimicrobial resistance (AMR) gene targets are selected to capture the majority of known sequence variants within each gene family. (C) BADLOCK diagnostic workflow for positive blood cultures. Following culture positivity, samples undergo heat lysis alongside Gram staining. Based on Gram-stain morphology, a gene panel is selected - in this case one that focuses on Gram-negative rods. A single master mix is split across RPA primer and CRISPR guide sets, each targeting a specific gene. Reactions are incubated for 50 minutes, and fluorescence readouts are used to determine target identity.

**Methods:**

BADLOCK couples recombinase polymerase amplification (RPA) with CRISPR-Cas13a detection in a one-pot, isothermal format. To simulate clinical samples, pooled human urine was spiked with 10⁵ CFU/mL of 30 uropathogenic isolates (five each of E. coli, K. pneumoniae, E. cloacae, C. freundii, and P. aeruginosa), alongside 13 isolates carrying AMR determinants (CTX-M-15, KPC-2, NDM-1, OXA-48). Multiple assay optimizations were tested, including template volume increase, RPA reagent adjustment, and lysis improvements. Final validation employed a spin-down plus heat lysis protocol followed by detection via fluorescent readout.

**Figure 3.**
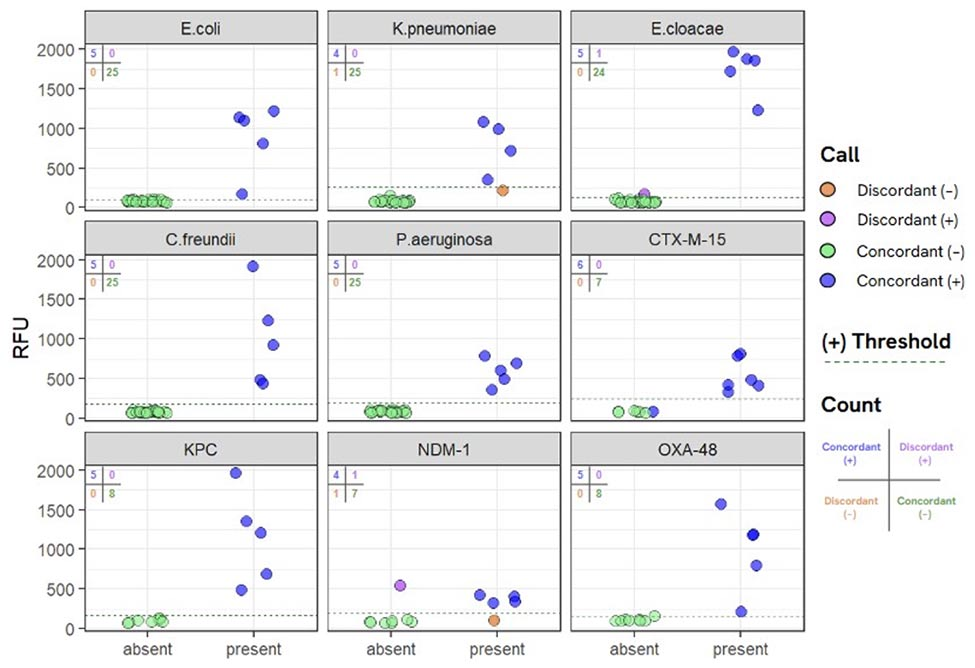
Validation of BADLOCK Diagnostic Panel for Bacterial Species and AMR Gene Detection in Urine Performance of the full BADLOCK panel for bacterial species identification and antimicrobial resistance (AMR) detection in mock urine samples. Fluorescence output (RFU) for each primer-guide pair tested across 30 mock urine samples spiked with either panel-positive or off-target bacterial isolates at 10^5^ CFU/mL. Each panel displays individual sample results for species-specific (E. coli, K. pneumoniae, E. cloacae, C. freundii, P. aeruginosa) and AMR-specific (CTX-M-15, KPC-2, NDM-1, OXA-48) targets. Points are color-coded by call type: concordant positive (blue), concordant negative (green), discordant positive (purple), and discordant negative (orange). Thresholds (dashed lines) were determined by calculating the mean RFU of off-target samples plus six times the standard deviation. Counts for each result type are shown in the top left of each subplot, organized in a confusion matrix.

**Results:**

Template volume and RPA reagent boosts yielded inconsistent gains. In contrast, sample concentration via centrifugation markedly enhanced signal across all targets without compromising specificity. Detergent-based lysis had minimal effect. The final workflow achieved 98.8% accuracy for bacterial species identification (163/165 correct) and 96.2% accuracy for AMR detection (50/52 correct). All discordant results were resolved upon retesting. Specificity across panels remained ≥ 87.5%, with most targets reaching 100%.

**Conclusion:**

This proof of concept study demonstrates that BADLOCK can accurately detect uropathogens and key resistance genes in urine within 1.5 hours. With further validation in patient-derived samples, this low-cost platform could support timely, targeted therapy for UTIs while mitigating the spread of AMR.

**Disclosures:**

All Authors: No reported disclosures

